# Editorial: Hernia related anatomy in abdominal wall surgery: Applied research in hernia repair

**DOI:** 10.3389/fsurg.2022.1031593

**Published:** 2022-09-26

**Authors:** Marko Konschake, Franz Mayer, Rene H. Fortelny

**Affiliations:** ^1^ Department of Anatomy, Histology and Embryology, Institute of Clinical and Functional Anatomy, Medical University of Innsbruck (MUI), Innsbruck, Austria; ^2^ Department of Surgery, Paracelsus Medical University, Salzburg, Austria; ^3^ Department of Surgery, General Hospital Hallein, Hallein, Austria; ^4^ Medical Faculty, Sigmund Freud Private University, Vienna, Austria

**Keywords:** surgery, hernia, applied anatomy, embryology, abdominal wall reconstruction (AWR)

**Editorial on the Research Topic** Hernia related anatomy in abdominal wall surgery: Applied research in hernia repair by Konschake M, Mayer F, Fortelny RH. (2022) Front. Surg. 9: 1031593. doi: 10.3389/fsurg.2022.1031593

This special issue of *Frontiers in surgery* concerning the research topic “Hernia Related Anatomy in Abdominal Wall Surgery” addresses research in the field of Applied Anatomy, Human Embryology and Hernia Surgery. All the original articles and reviews presented are dealing with important facts with the goal of placing its findings to an interested broader surgical community.

**Figure 1:**
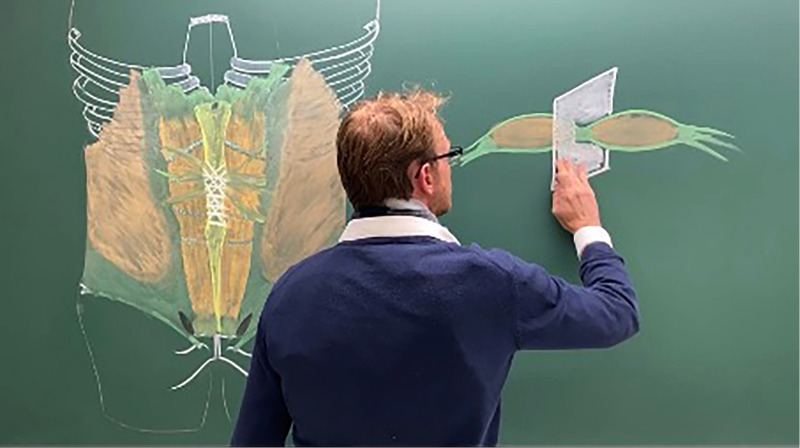
Prof. Dr. Yohann Renard, Department of Surgery, Department of Anatomy, University Hospital Reims (France).

Without having detailed knowledge in anatomy surgeons will quickly get into troubles. As this kind of knowledge usually is acquired during university studies, there is a strong need to transpose this knowledge later into clinical practice. Doing this needs clinical mentors being well trained in teaching Anatomy or dedicated post-graduate courses to specific fields of surgery – such as the Hernia Compact Course of The German Hernia School (1) in Abdominal Wall Reconstructive (AWR) Surgery – with the help of specialists in Applied Anatomy, who know to “speak the language of clinicians”. Beside that it needs surgeons, who have deep insights in the science of Anatomy. Yohann Renard, a surgeon and anatomist at the university of Reims (France), can be denominated as being a perfect representative of a handful of surgeons worldwide who are highly competent in explaining detailed anatomical knowledge to clinicians (Figure 1). By writing a paper to this issue in *Frontiers of Surgery*, Andreas Lorenz, a surgeon at the university of Innsbruck (Austria), has imparted detailed anatomical knowledge of the preperitoneal space, the transversalis fascia and the urogenital fascia - which are of highest relevance for many different techniques in incisional hernia repair and minimally invasive techniques in groin hernia surgery - into the surgical community. Another issue of prevention is knowledge of neuroanatomy especially in the groin as recently published by our Austrian Hernia Group (2), which established an annual workshop regarding this topic.

In recent years, surgery of the abdominal wall has evolved massively with new techniques especially various techniques for myofascial-release of the abdominal wall, such as transversus abdominis release (TAR). New insights to the operation field (minimal-invasive or robotic techniques) require new ways for access to anatomical layers needing special anatomic training for example by cadaver workshops. In order to avoid complications, such as injuries to nerves and vessels, but also unphysiological separations of musculoaponeurotic structures, anatomical knowledge is required to a special degree. The contributions of this publication are therefore essential to expand this knowledge.

This issue comprises highly qualified papers, which provide interesting findings in human embryology of the anterior abdominal wall and associated malformations. These articles will lead to a better understanding of the development of the abdominal wall, possible herniations and therefore of surgical repair of abdominal and diaphragmatic pathologies.
